# Feasibility of weekly participant-reported data collection in a pragmatic randomised controlled trial in primary care: experiences from the BATHE trial (Bath Additives for the Treatment of cHildhood Eczema)

**DOI:** 10.1186/s13063-018-2962-3

**Published:** 2018-10-24

**Authors:** Beth Stuart, Kate Rumsby, Miriam Santer, Matthew J. Ridd, Nick A. Francis, Maria Chorozoglou, Carla Spreadbury, Mary Steele, Claire Nollett, Lyn Liddiard, Martina Prude, Julie Hooper, Emma Thomas-Jones, Amanda Roberts, Kim S. Thomas, Hywel C. Williams, Paul Little

**Affiliations:** 1 Department of Primary Care and Population Sciences, University of Southampton, Aldermoor Health Centre, Aldermoor Close, Southampton, SO16 5ST UK; 2 School of Social and Community Medicine, University of Bristol, Bristol, UK; 3 Wales School of Primary Care Research, Cardiff University, Cardiff, UK; 4 Southampton Health Technology Assessment Centre (SHTAC), University of Southampton, Southampton, UK; 5 Centre for Clinical and Community Applications of Health Psychology, Psychology, Faculty of Social and Human Sciences, University of Southampton, Southampton, UK; 6 Centre of Evidence-Based Dermatology, University of Nottingham, Nottingham, UK

**Keywords:** Feasibility, Repeated measures, Trial methodology, Eczema, Atopic dermatitis, POEM, PROMs

## Abstract

**Background:**

Patient-reported outcomes measures in clinical trials ensure that evaluations of effectiveness focus on outcomes that are important to patients. In relapsing-remitting conditions, such as eczema, repeated measurements may allow a more accurate reflection of disease burden and treatment effect than less frequent measurements.

We asked parents/carers of children with eczema taking part in a trial of bath emollients to complete weekly questionnaires for 16 weeks.

**Methods:**

The objective of this study was to determine the acceptability and practicality of collecting weekly measures of eczema severity online for 16 weeks in children aged 1 to 11 years as part of the BATHE study.

BATHE randomised patients to bath emollients plus standard eczema care or standard eczema care only. The primary outcome was eczema severity, measured by the seven-item Patient-Oriented Eczema Measure (POEM) repeated weekly for 16 weeks. Acceptability was explored through qualitative interviews with 10 participants. Interviews were audio-recorded, transcribed and analysed thematically. Practicality was assessed by exploring the completeness of the data and keeping a log of any problems.

**Results:**

Four hundred and eighty-two participants were recruited to the trial and 429 opted to complete measures online (89.0%). Data were collected online for 83% of time points over the 16-week period and there was no association between socio-demographic characteristics and data completeness. Two hundred and six (48%) completed their weekly data every week for 16 weeks and 341 (79%) completed it at least 80% of the time. The mean number of weeks completed was 13.3 out of 16 (SD 4.2).

Interviewees said that they understood the rationale behind weekly collection and some welcomed this as it helped them realise how their child’s eczema changed weekly. Whilst some interviewees spoke of weekly questionnaires as onerous, others said that they found them quick and easy. Reminders were welcomed.

Parents/carers seemed happy to receive telephone reminders and it was sometimes useful for eliciting problems relating to obtaining trial medication or password problems for online data collection.

**Conclusions:**

Amongst this population, high levels of data completeness suggests that weekly completion of the online questionnaire appears to be acceptable and feasible over a 16-week period.

**Trial registration:**

ISRCTN84102309. Registered on 9 December 2013.

## Background

Repeated measures are frequently used in clinical trials, rather than a single endpoint, in order to allow researchers to explore how treatment or the trajectory of a condition changes over time [1]. Repeated measures also allow for increased power due to the reduced intra-participant variability, which results in smaller sample size requirements [1, 2]. Whilst regular measurements may help to give a more accurate impression of changing disease states, this must be balanced against the feasibility of doing so within a trial context and the burden it places upon participants and research staff to ensure that these measures are completed.

Eczema is a common condition in childhood and can have a significant impact on quality of life for both children and parents due to itching and disturbed sleep [3]. In order for interventions to improve the quality of life in a way that is relevant to children and parents, it is important that the outcome measures used in studies capture their experiences of eczema and any perceived benefits of treatments Patient-reported outcome measures (PROMS) are reported directly by participants and are being used increasingly as primary outcomes in clinical trials in order to assess the overall patient experience or perception of benefit to participants [4].

Capturing the experiences of people with eczema is complicated by the relapsing and remitting nature of the condition. Therefore, gathering information regularly over time is essential for understanding disease burden [5], and to accurately assess the impact of interventions.

The BATHE trial aimed to assess the clinical and cost effectiveness of bath emollients for eczema [6]. The primary outcome was the Patient Orientated Eczema Measure (POEM) measured weekly for 16 weeks. POEM is the patient-reported outcome measure recommended by NICE [7] and the international Harmonizing Outcome Measures for Eczema initiative (http://www.homeforeczema.org/) for measuring eczema symptoms [8].

Although there are good reasons for collecting POEM scores on a weekly basis from participants, prior to the start of this trial there was no evidence as to whether it would be acceptable or practical to do so. The study team had experience of two prior studies which had gathered data from participants on a daily basis. The Softened Water Eczema Trial (SWET) had gathered POEM daily data via paper diaries [9]. The data were very complete with 94% of all data points complete at 16 weeks [10]. However, with paper diaries there is a risk that participants may complete the measures at a later date, backfilling the diary in order to return complete data to the research team but increasing the risk of recall bias [11]. An observational study of flare triggers also recorded eczema on a daily basis but using electronic diaries [12]. The diary could not be completed after midnight on any given day, thereby eliminating the risk of backfilling the diary, but the data were much less complete, with only 60% of the data complete at 16 weeks [10].

The BATHE trial aimed to reduce the burden by collecting the POEM scores weekly via an online platform. This paper reports on the acceptability and practicality of weekly data collection from participants in the context of an eczema trial.

## Methods

### The BATHE study

The BATHE trial was a pragmatic, unmasked, two-armed randomised controlled trial in general practices in England and Wales. Children aged 12 months to 11 years who met the UK Diagnostic Criteria for Eczema [13], and who had mild to moderate eczema according to the Nottingham Eczema Severity Score [14], were randomised to either standard care or bath emollients plus standard care. Participants were recruited from 96 practices in Wales, South of England, West of England and Wales. Further details regarding the trial, including full inclusion and exclusion criteria, can be found in the published protocol [6] and published study results [15].

The primary outcome was the Patient Orientated Eczema Measure (POEM) [16] completed weekly for 16 weeks. POEM is a patient-reported outcome based on symptoms experienced over the previous week, which can be completed by the child or their parent / carer. POEM includes seven questions about eczema symptoms over the previous week that are summed to give a score from 0 (eczema is clear or causing no impact) to 28 (very severe eczema). POEM is recommended by NICE [17] and the international HOME initiative (Harmonising Outcome Measures in Eczema) and is the only patient-reported outcome that demonstrated sufficient validity and repeatability in a systematic review of outcome measures for eczema [18, 19]. Our primary outcome measure was based on repeated measures of POEM data collected weekly over 16 weeks because this reflected the impact of this relapsing and remitting chronic condition better than comparing outcomes at a single follow-up time point. In addition to collecting the POEM each week, we also asked a weekly question about adverse events. Parents were encouraged to complete measures online and offered the option to complete on paper only if they stated that they felt online completion would be difficult.

Following the baseline appointment there were no other face-to-face study visits, so efforts were made from the outset to ensure that participants remained engaged with the trial. These included small gifts (a bath duck branded with the study logo, post-it notes and a bendy eraser) given to each child at the start of the study. Communication was encouraged by setting up a study website, posting out birthday cards for the children and Christmas cards for the families, as well as quarterly newsletters. There were also incentives to encourage continued participation including a ‘thank you’ card and £10 voucher sent to parents before the 16-week questionnaire was due, and all participants were eligible for inclusion in a prize draw for a tablet computer at the end of the study.

### Sample size

The sample size for BATHE was calculated for repeated measures analysis of covariance (ANCOVA) in weekly POEM scores over 16 weeks. With alpha 0.05 and beta 0.1, we aimed to detect a difference of 2 points in the POEM over the 16-week period (SD 7.0) based on data from the SWET trial [9]. This gave a sample size of 338, rising to 423 with a 20% allowance for loss to follow-up. Early data suggested that approximately 80% of participants in both groups were adherent to treatment allocation. As we were keen to report a secondary per-protocol analysis in addition to a primary intention-to-treat analysis, we revised our sample size to reflect this with approval from the funder, sponsor and Trial Steering Committee. Assuming that 80% of participants adhere to treatment allocation, we required an additional 68 participants to retain 90% power for the per-protocol analysis. This gave an increased recruitment target of 491 participants.

The primary analysis for the trial used a mixed model (for repeated measures), allowing participants to contribute data for all the time points for which they have completed a weekly questionnaire. For this study, we have used all available data.

### Data collection

Participants received a unique ‘log in’ and entered their data online into a validated database. Parents/carers were notified that the weekly questionnaire was available to complete by email and by text/SMS on the day that it fell due. A reminder was sent 2 days later if the POEM had not been completed. The online questionnaires remained available for completion for a further 5 days, when they were replaced by the following week’s questionnaire. There was no facility to complete the online POEM questionnaire retrospectively once the 7-day period had elapsed.

Parents/carers had contact with the same member of staff for the duration of the study so far as possible. Questionnaire completion was monitored by the trial team and efforts were made to contact participants by telephone if their completion rate fell below 80%. Particular efforts were made to speak to those who failed to complete the first week’s POEM, as this would suggest difficulties with the log-in system. Particular efforts were also made to speak to parents/carers who had not completed the 16-week questionnaire.

Although the online portal was designed to be accessed via a desktop computer or tablet, we suspected that many parents would access the website via their mobile phone. Data on the user agent and referrer were automatically captured by the online platform, enabling us to describe the type of device from which parents/carers logged in to record the weekly data.

### Quantitative analysis

The proportion of parents/carers who opted for online completion and the completeness of the data, both in terms of the number of weeks, on average, for which they completed measures and the overall number of data points collected from the study population have been analysed descriptively and graphically. Associations between key participant characteristics and data completeness were explored using chi-squared tests with a 5% significance level.

### Qualitative interviews

All parents of affected children who were assigned to the Southampton trial centre (across three geographical counties in the South of England) within the first 10 weeks of the trial were contacted to offer an interview primarily by email, then by telephone if there was no email response. An answerphone message was left for the first occasion of an unanswered call, but no further messages were left in the further calls that were made (up to three per participant).

Semi-structured interviews were undertaken with 10 parents following an interview guide. Interviews were carried out either face-to-face or, where the participant preferred, by telephone. Interviews were audio-recorded, professionally transcribed and transcripts were checked against recordings. An inductive thematic analysis [20] was conducted to explore people’s experiences and views of participating in the BATHE study.

Two authors (CS and MS) read the transcripts several times and produced a coding framework, which developed iteratively as further transcripts became available. Disconfirming cases were sought. Data saturation was not achieved for all themes (e.g. motivation for participating in trials) but was achieved for our main theme exploring experiences of weekly data collection. Pseudonyms have been applied in reporting the data and for all quotes used below.

## Results

Four hundred and eighty-two participants were recruited from 96 general practice (GP) surgeries across three centres: Southampton, Bristol and Cardiff (South of England, West of England and Wales). Only four participants withdrew from the trial. Invitation letters were posted from the participating GP surgeries to 12,523 children who had received a prescription for eczema treatment within the previous 12 months. The mean response rate overall was 11.55% (3.9–25%): positive eligible replies, after exclusions, averaged 3.86% (0–14.3%) of invitations sent.

### Practicality

Online weekly questionnaire completion was chosen by 429 (89.6%) participants. Of the remaining 50 participants, 27 (5.6%) requested paper and 23 (4.8%) were switched from online to paper format after discussion with the study team. Reasons for this primarily related to technical issues: some parents became discouraged after having problems logging in to the online database or issues with connectivity. Where parents/carers contacted the study team about this issue, they were often able to log in once their password had been re-set, indicating that they had forgotten their log-in details (which were case-sensitive). However, it is unclear how many gave up at this point without reporting the problem.

Log-in problems were occasionally compounded by a failure to understand the automated nature of the system and that they would be unable to go back to complete past POEMs. Some parents reported difficulties getting access to the family computer and there were also individual issues such as changing service providers, poor Internet connections, as well as long family holidays. Paper case report forms (CRFs) consisted of four POEMs printed in A5 booklets. They were posted out monthly, with a covering letter and pre-paid envelope.

The characteristics of the participants are set out in Table 1. Although the numbers completing on paper were small compared to those completing online, there was no evidence of any difference in characteristics by method of completion.

**Table 1:** Participant characteristics by method of completion

	Online (*n* = 429)	Paper (*n* = 27)	Switched to paper [23]
Child age in years (SD)	6.6 (2.8)	6.3 (2.6)	6.2 (2.6)
Child gender			
Male	206 (48.1%)	16 (59.2%)	15 (68.2%)
Female	222 (51.9%)	11 (40.7%)	7 (31.8%)
Ethnicity			
White	364 (86.1%)	23 (85.2%)	16 (72.7%)
Asian	16 (3.8%)	1 (3.7%)	1 (4.6%)
Chinese	8 (1.9%)	1 (3.7%)	2 (9.1%)
Black	11 (2.6%)	0	0
Mixed	21 (5.0%)	2 (7.4%)	3 (13.6%)
Other	3 (0.7%)	0	0
How cost of living affects household			
Find it a strain	11 (2.6%)	1 (3.7%)	2 (9.1%)
Have to be careful	164 (39.0%)	13 (48.1%)	8 (36.4%)
Able to manage	168 (39.9%)	11 (40.7%)	11 (50.0%)
Quite comfortable	68 (16.2%)	2 (7.4%)	1 (4.6%)
Not answered	10 (2.4%)	0	0

**Fig. 1:**
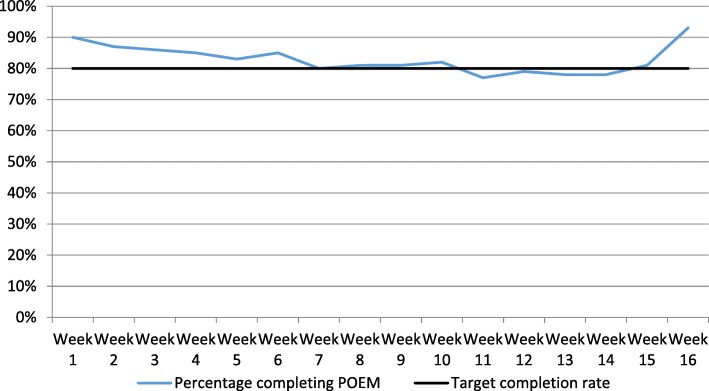
Percentage of participants completing the Patient-Oriented Eczema Measure (POEM) each week

As shown in Fig. 1, the online weekly POEM was well completed. If all 429 participants completed their data as expected, we would have collected 6864 data points. We collected 5680 data points, making the data 83% complete. Only four participants were lost to follow-up, due to withdrawal from the study. The primary analysis of the trial was, therefore, able to include data from 99% of the participants and was over 80% complete. Given that the sample size calculation allowed for 20% of participants to be lost to follow-up, this was well within what was required in order to provide adequate data for the analysis of the primary outcome.

There was a slight increase in the percentage completed at week 16, despite the final POEM being embedded within a longer questionnaire. Parents were sent a ‘thank you’ card and a £10 voucher just before the 16-week questionnaire was due, and a reminder letter and paper copy of the questionnaire were posted out if the online questionnaire had not been completed within 7 days (*n* = 80).

Of the 429 parents/carers completing their data online, 206 (48%) completed their weekly data every week for 16 weeks. Three hundred and forty-one (79%) completed their weekly data at least 80% of the time. The median number of weeks completed was 15 out of 16 (inter-quartile range (IQR) 13, 16). There were no differences between the two trial arms with respect to completeness with a median of 15 [12, 16] for the group allocated to the usual-care arm and a median of 15 (IQR 13, 16) for those allocated to the bath-emollient arm. Of those who completed online, 93 (49%) in the usual-care arm and 109 (47%) in the bath-emollient arm, completed their weekly measures for all 16 weeks.

**Fig. 2:**
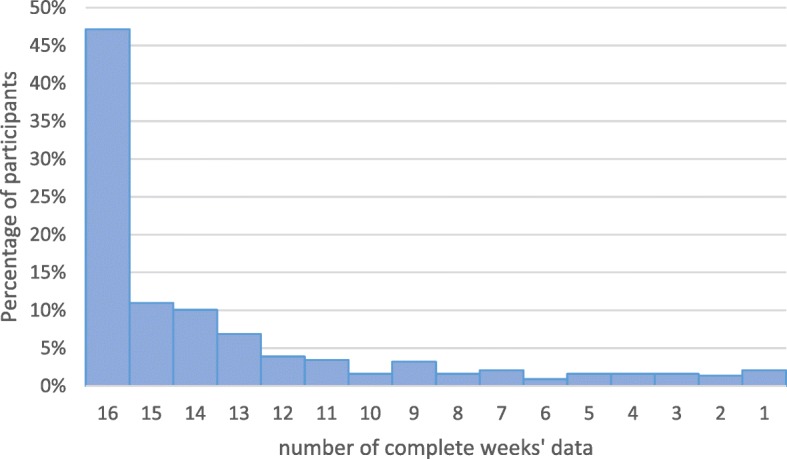
Data completeness over the 16-week period

There was no association between study arm, sex of child, age of child, ethnicity or financial circumstances and the completeness of the data. (see Table 2). Data from the group who completed on paper were less complete with only 42% of weekly data points completed. Figure 2 shows the distribution of responses over the 16-week study period.

**Table 2:** Data completeness by participant characteristics

	Data complete at least 80% of the time (*n* = 341)	Data not complete at least 80% of the time (*n* = 88)	Chi-squared test statistics (*p* value)
Study arm			2.86 (*p* = 0.104)
Usual care	145 (43.2%)	46 (52.9%)	
Bath emollient	191 (56.6%)	41 (47.1%)	
Child age			2.86 (*p* = 0.091)
Under 5 years	112 (32.8%)	37 (42.5%)	
5 years and over	229 (67.2%)	50 (57.5%)	
Child gender			0.07 (*p* = 0.787)
Male	163 (47.8%)	43 (49.4%)	
Female	178 (52.2%)	44 (50.6%)	
Ethnicity			3.46 (*p* = 0.063)
White	297 (87.6%)	67 (79.8%)	
Non-white	42 (12.4%)	17 (20.2%)	
How cost of living affects household			3.06 (*p* = 0.382)
Find it a strain	8 (2.4%)	3 (3.8%)	
Have to be careful	139 (41.9%)	25 (31.7%)	
Able to manage	131 (39.5%)	37 (46.8%)	
Quite comfortable	54 (16.3%)	14 (17.7%)

The webpages where parents/carers completed the POEM were designed to be completed online on a computer or laptop. They could be viewed and completed on a mobile phone but were not designed with this in mind. However, of the 9784 times that parents logged in to the website, 5963 (61%) were from mobile devices. A further 912 (9%) were from tablets whilst 2909 (30%) were from computers or laptops.

Feedback from trial staff suggested that the collection of weekly data was not problematic. On average, they reported needing to make a total of 6–10 telephone contacts per week to remind parents to complete the POEM online. Overall, approximately 25% of the sample required following up by telephone at some point in order to ensure that their measures were completed. Over the course of the study, 274 (57%) had no direct contact with the trial team (beyond automated messages, newsletters, etc.). Thirty-four had contact re issues with prescriptions/medications/reactions, and 71 had contact re technical issues. The technical problems were the most burdensome to deal with.

Trial staff felt that the efforts that had been made to engage participants with the study were helpful in ensuring that the weekly measures were completed. Trial staff also believed that parents having a single point of contact within the trial team throughout the study also encouraged engagement.

### Acceptability

Nineteen parents/carers were contacted to request a qualitative interview, of whom 10 were interviewed: none refused but seven did not respond to repeated telephone calls to try to arrange an interview. A further two interviews were arranged, but were cancelled by the participant. Five interviews were carried out face-to-face (four in the interviewees’ homes, and one in a public setting) and five interviews were carried out by telephone. All those interviewed were mothers, aged 32–49 years with a child aged between 2 and 8 years old.

Almost all interviewees said that they found the questionnaires quick and easy. A few mentioned that they did not find questionnaires difficult but were pleased that they would become less frequent as the trial progressed (the POEM was asked monthly between week 16 and week 52).

Annabel gives a very typical answer:

Interviewer: ‘What do you think of the weekly questionnaires?’

Annabel: ‘Fine…to the point – quick – no problem…’*.*

Most interviewees said that they understood why the questionnaires were weekly as their child’s eczema was variable and some actually liked completing the questionnaires as it made them appreciate this variation more. One person reported initially thinking that weekly questionnaires were too much, as she did not think that her child’s eczema severity would alter. During the interview, however, Louisa reports that she was able to notice differences on a weekly basis:

Louisa: ‘ When I heard that it was going to be weekly, I thought “well, surely things aren’t going to change that much”, but actually, when it comes to it – my little one’s skin, it really does change that much…So I think weekly is a good interval’.

One interviewee reported finding difficulty with the weekly questionnaires, as she was forgetting to complete them:

Interviewer: ‘…What have you thought of the weekly questionnaires?’

Annie: ‘I’ve had trouble doing it every week: yes, I look at them and then I – put my phone down and then I forget all about it…It’s remembering…’*.*

In addition to their feelings about weekly questionnaires for the first 16 weeks, parents/carers were asked about their feelings around the year-long nature of the trial. (Participants continued to receive monthly questionnaires after 16 weeks until 52 weeks). A number of interviewees reported seasonal variation in their child’s eczema, and thought that the year-long follow-up would allow this to be reflected in the data:

Linda: ‘You need a year – I would have thought…Our (eczema) changes in every season, depending on whether we’ve got the heating on…’.

Interviewer: ‘So the fact it is... ’.

Linda: ‘It is a year, it’s brilliant’.

## Discussion

We found that it is both feasible and acceptable to collect repeated measures of eczema severity online over a 16-week period in the context of a clinical trial. Parents/carers and trial staff gave positive feedback about their experience of completing the measures online. Some participants did have difficulties, and 23 had to be switched to paper completion. However, almost 90% of participants completed their data online, the data were 83% complete and only four participants were lost to follow-up, allowing 99% of participants to contribute at least one data point to the primary analysis. There was no association between participant socio-demographic characteristics and data completeness.

BATHE was a pragmatic trial and it was not possible to blind participants to their group allocation. This led to concerns that there might have been differential attrition, with participants randomised to usual care feeling less engaged and failing to complete measures, which may lead to biased results [21]. However, we did not find evidence of this. The median number of weekly measures completed was 15 out of 16 weeks in both arms, suggesting that strategies to keep participants in both groups engaged with the study were successful.

There was evidence that although data completion started high, with over 90% responding in the first week, the response rate as shown in Fig. 1 declined over time, falling to just below 80% in weeks 11–14 of the study. The trial design meant that resources were targeted at the final follow-up point, when a number of other measures were due and this increased the completion rate at week 16 to 93%. Targetting resources at key time points, particularly during longer follow-up periods, may help to keep participants engaged with the study and increase the completenss of the data.

Although rates of data completion were high, the trial’s data collection procedures may not have been optimised for the methods that participants used. Whilst the data collection was designed primariliy to be completed online via a computer or tablet, 61% of log ins were from mobile devices. According to the Office for National Statistics, Internet access ‘on the go’ using mobile devices has increased steadily over the last 5 years with 73% of adults accessing the Internet from a mobile phone in 2017 [22]. Future studies should acknowledge this trend and ensure that the data collection procedure is optimised for viewing and completion on a mobile device.

Whilst using an online data collection approach did yield relatively complete data in this study, there were challenges. Difficulties with logging in were one of the main reasons that participants contacted trial staff. Log-in problems, including lost IDs and passwords, may have resulted in some participants being put off taking part without further contact with the trial team. In future, other secure log-in options, such as an app or an encrypted link for each participant, might help to avoid this potential difficulty.

### Strengths and limitations

This study only evaluated the feasibility and acceptability of completing the weekly POEM online. As noted above, digital technologies and mobile phones are becoming increasingly pervasive in everyday life, and, in future, people may find it more convenient to use apps or SMS to provide data [23] Participants did receive text message reminders but could not complete their POEM questionnaire via text. Although we believe that it is very likely that the feasibility of completing the POEM weekly online would translate into these other media, this would have to be evaluated further [24].

Qualitative interviews with a larger number of participants, including participants who completed paper questionnaires, or purposively sampling participants with low completion rates, may have generated further useful insights and is planned for future trials.

### Findings in context of existing research

The collection of repeated measures data is known to suffer from problems with attrition [25] as is data collected as part of studies administered online [26]. The level of completeness achieved with the online weekly POEM of 83% was comparable to that observed in three other recent eczema trials. The COMET feasibility study asked participants to complete a daily diary for 12 weeks by paper or using an electronic app. Due to problems with the online app, only 11/196 participants completed measures using the app but POEM data were complete for between 70 and 75% of all participants (paper and online) over the 12-week period [27]. The CREAM trial measured POEM data at 4 weeks and 3 months and achieved a response rate of 86.7 and 65.5%, respectively [28]. The CLOTHES trial collected POEM data weekly for 24 weeks and 85% of participants completed 12 or more of the weekly questionnaires [29].

Weekly data collection has been used in clinical trials of other medical conditions. A trial of weekly self-monitoring in asthma concluded that it improved asthma control and was feasible, with 80% completing the weekly data collection online as instructed during the first 3 months [30]. Self-monitoring is frequently included as part of behavioural weight-loss interventions [31] and several studies have included weekly diaries to collect physical activity and nutrition information. These studies have found weekly diaries to be reasonably complete – 62.7% in Carel et al.’s 14-week randomised controlled trial (RCT) [32] and 83% in the first 6 months of Wing et al.’s 18-month RCT [33]. Weekly testing has also been trialled for the self-management of oral anti-coagulant therapy and showed that weekly self-management is feasible and provided some protection from complications [34]. This study, therefore, fits within existing literature suggesting that weekly data collection can be feasible and acceptable in a clinical trial setting.

### Implications for future research

It is possible that frequent assessment allows participants to provide more accurate recall and thus reduces bias [35]. However, it is also possible that repeatedly asking participants about eczema symptoms changes their perception and hence their responses, causing a response shift [36]. It may alter adherence to study medications and co-treatment and introduce a performance bias if weekly assessment of eczema status is not part of normal routine care. It is, therefore, important to evaluate whether, in addition to being feasible and acceptable, the collection of weekly POEM data is actually useful when compared to using measures taken at less frequent time points. The HOME group is continuing to explore the best way to capture long-term control of eczema [37] and the data gathered as part of the BATHE trial will be evaluated alongside data from other eczema trials to help to inform decisions about how frequently to ask participants to complete the POEM in future studies and over what time period.

## Conclusion

In this population, weekly completion of the online questionnaire appeared to be acceptable and feasible over a 16-week period. Email, text and telephone reminders seemed helpful to most participants.

## Abbreviations

ANCOVA: Analysis of covariance
BATHE: Bath Additives for the Treatment of cHildhood Eczema
CRF: Case report form
HOME: Harmonising Outcome Measures in Eczema
IQR: Inter-quartile range
POEM: Patient-Oriented Eczema Measure
PROM: Patient-reported outcome measure
RCT: Randomised controlled trial
SWET: Softened Water Eczema Trial
